# Sequential Fermentation in Red Wine *cv*. Babić Production: The Influence of *Torulaspora delbrueckii* and *Lachancea thermotolerans* Yeasts on the Aromatic and Sensory Profile

**DOI:** 10.3390/foods13132000

**Published:** 2024-06-25

**Authors:** Stipe Ivić, Ana Jeromel, Bernard Kozina, Tihomir Prusina, Irena Budić-Leto, Ana Boban, Višnja Vasilj, Ana-Marija Jagatić Korenika

**Affiliations:** 1 Institute for Adriatic Cultures and Karst Reclamation, Put Duilova 11, 21000 Split, Croatia; stipe.ivic@krs.hr (S.I.); irena.budic-leto@krs.hr (I.B.-L.); aboban@krs.hr (A.B.); 2 Faculty of Agriculture, University of Zagreb, Svetošimunska cesta 25, 10000 Zagreb, Croatia; amajdak@agr.hr (A.J.); bkozina@agr.hr (B.K.); 3 Faculty of Agriculture and Food Technology, University of Mostar, Biskupa Čule 10, 88000 Mostar, Bosnia and Herzegovina; tiho@vinarija-citluk.ba (T.P.); visnja.vasilj@aptf.sum.ba (V.V.)

**Keywords:** aroma profile, gas chromatography–mass spectrometry, non-*Saccharomyces* yeast, sensory properties, volatile compounds

## Abstract

This research aimed to analyze the impact of two different non-*Saccharomyces* yeast species on the aromatic profile of red wines made from the *cv.* Babić (*Vitis vinifera* L.) red grape variety. The grapes were obtained from two positions in the Middle and South of Dalmatia. This study compared a control treatment with the *Saccharomyces cerevisiae* (*Sc*) strain as a type of sequential inoculation treatment with *Lachancea thermotolerans* (*Lt x Sc*) and *Torulaspora delbrueckii* (*Td x Sc*). The focus was on the basic wine parameters and volatile aromatic compound concentrations determined using the SPME-Arrow-GC/MS method. The results revealed significant differences in *cis*-linalool oxide, geraniol, neric acid, and nerol, which contribute to the sensory profile with floral and rose-like aromas; some ethyl esters, such as ethyl furoate, ethyl hexanoate, ethyl lactate, ethyl 2-hydroxy-3-methylbutanoate, ethyl 3-hydroxy butanoate, diethyl glutarate, and diethyl succinate, contribute to the aromatic profile with fruity, buttery, overripe, or aging aromas. A sensory evaluation of wines confirmed that *Td x Sc* treatments exhibited particularly positive aromatic properties together with a more intense fullness, harmony, aftertaste, and overall impression.

## 1. Introduction

The most recent trend in enology involves the utilization of selected non-*Saccharomyces* (non-*Sc*) yeasts. Non-*Sc* yeasts encompass any yeast species found in wine other than *Sc* that have a beneficial impact on the winemaking process. More attention was paid to them in winemaking due to their influence on wine aroma and polyphenolic composition [1]. In the past, yeast selection for winemaking was limited to the *Saccharomyces cerevisiae* species (*Sc*). However, now there are numerous commercial yeast strains derived through specific genetic methods and selection [2]. These strains can produce varying concentrations of secondary compounds, which contribute to the unique characteristics of wine. While *Sc* remains the dominant species for alcoholic fermentation, non-*Saccharomyces* yeasts have gained interest in recent years [3,4]. Traditionally, non-*Sc* yeasts have been considered as contaminants in winemaking, and measures such as must pasteurization, sulfite addition, and equipment and processing area disinfection have been routinely employed to eliminate them from the fermentation process. Due to their limitations, such as their sensitivity to ethanol and SO_2_, non-*Sc* yeasts can only be involved in sequential fermentation with *Sc* [5]. This method closely resembles spontaneous fermentation. The incorporation of non-*Sc* yeasts in the winemaking process yields several advantageous effects on wine quality, including moderate ethanol levels, an increased glycerol content, a higher acidity, and a more intricate aromatic profile. For this study, two commercially available non-*Saccharomyces* yeast strains were used—*Torulaspora delbrueckii* (*Td*), the oldest, and *Lachancea thermotolerans* (*Lt*), the most recently selected [6]. *Lt* is renowned for acidifying musts with low total acidity and high pH values [7]. Nowadays, it is widely employed in sequential inoculation for red wine vinification through the production of significant concentrations of lactic acid. This inherent feature renders it a valuable resource for blending and/or re-equilibrating red wines from warm climates [8] like *cv.* Babić, an autochthonous variety of red grapes, cultivated in the warm, coastal region of Dalmatia, in Croatia. In addition to enhancing freshness and acidity, *Lt* contributes to the aromatic complexity right from the beginning of alcoholic fermentation. *Torulaspora delbrueckii* is also recommended for the modification of the aroma profile of wine. Its metabolic activity facilitates the release of terpene aromas, including α-terpineol and linalool [9]. The utilization of this strain can intensify the fruity characteristics of wines. Furthermore, it can enhance red wine color, reduce ethanol levels, decrease fatty acid concentrations, and increase mannoprotein and glycerol through sequential fermentation with *Sc* [10]. So, sequential fermentation with different yeast strains enhances wine’s flavor profile by increasing the presence of diverse volatile compounds such as alcohols, esters, phenols, terpenes, and C13-norisoprenoids [11].

The fermentation process of must or pomace plays a pivotal role in extracting aromatic compounds from grapes. This extraction process alters the aromatic compounds and generates secondary metabolites produced by yeast. Moreover, the presence of other compounds in wine, such as ethanol, phenols, and acids, also impacts the composition of volatile components and thus influences the aroma perceived in a glass of wine [12]. The perception of volatile compounds in wine aroma is closely linked to the orthonasal and retronasal human senses. The complexity of wine aroma arises from the interaction between volatile compounds and other components such as water, ethanol, phenolic compounds, and polysaccharides. Organic acids have various important functions in the context of wine production. They contribute significantly to the wine’s overall stability, both from a microbiological and physicochemical standpoint [13]. Furthermore, these acids have a notable impact on the wine’s visual perspective such as color intensity, as well as aging potential and flavor balance [14]. Additionally, organic acids influence the oxidation process of compounds found in both the must and the final product, as well as microbial metabolism, protein and polysaccharide solubility, potassium bitartrate solubility, and the efficacy of sulfur dioxide, fining agents, and pectolytic enzymes [14].

This study aimed to examine the impact of two different non-*Sc* yeast species on the aromatic and sensory characteristics of red wines produced from the Babić grape variety planted in two vine-growing positions.

## 2. Materials and Methods

### 2.1. Sample Preparation

The *cv*. Babić grapes were cultivated in the warm vine-growing hills of Šibenik (locality Jadrtovac) and Primošten (locality Široke) in the sub-regions (Protected Designation of Origin) of Middle and South Dalmatia. In the Jadrtovac locality, 50 ha of vineyards, at 70 to 100 m above sea level, mainly under the *cv.* Babić, were planted in the period from 2007 to 2012. The direction of the rows is north–south. The trellis system was a modified cordon with two cuttings with two to three buds. The soil is brown and skeletal. The position is extremely windy, and the entire production is organic with a maximum of two treatments per season with sulfur and copper preparations. The Široke locality is located on the southern slopes, between 230 and 250 m above sea level, characterized by natural rock material ingrown into the soil, which must be extracted manually to acquire a small quantity of soil in the lot (cassette) for cultivation. The direction of the rows is west–east. The total area of the vineyard is about 0.23 ha with about 1250 vines, and is more than 50 years old. The cultivation form was ‘en goblet’. This form of cultivation produces 4–7 bunches per vine, but it is important to note that the wind is one of the most important characteristics of this terroir and that part of the crop is regularly lost due to the strong winds.

The research specifically concentrated on the vintage 2020. The grape harvest was performed manually during the early morning hours, with each site yielding quantities of 200 kg, grapes were transported in plastic crates with a capacity of 20 kg, and primary processing was immediately performed, involving destemming and crushing. An electric crusher-destemmer was used for the primary processing of the grapes. The experiment was designed with three treatments for each of the two wine-growing positions with each treatment comprising three replications (Table 1).

### 2.2. Alcoholic Fermentation Trials

Detailed protocols for alcoholic fermentations are presented in Table 2. The control treatment (K) pomace was treated with 10 g/hL of K_2_S_2_O_5_, and 2 g/hL of pectolytic enzyme. Micronutrient-rich inactive yeast was added to the pomace, prepared in rehydration water with selected *Sc* yeast. Next, 72 h into fermentation, a complex yeast nutrient was added.

The *Lt x Sc* and *Td x Sc* musts were treated with 3 g/hL of K_2_S_2_O_5_ and 2 g/hL of pectolytic enzyme. Treatment *Lt x Sc* was inoculated with rehydrated *Lt* strains, and yeast nutrients were added. The pomace was sequentially inoculated 72 h into fermentation with the rehydrated *Sc* strain. Treatment *Td x Sc* was inoculated with rehydrated *Td* strains, yeast nutrients were added, and the pomace was inoculated with rehydrated *Sc* yeast strains 72 h into alcoholic fermentation.

All treatments were punched down daily, every eight hours, with the pomace temperature ranging from 22 to 25 °C. After eight days of maceration, the pomace was pressed and the partly fermented must was transferred to 10 L glass carboys (three replications per treatment) at 22 °C. During the alcoholic fermentation process, the degradation of sugar was monitored daily using a refractometer and specific gravity, and the must temperature was measured. One month after the first racking, wines from all replications were sampled for their chemical composition analysis.

### 2.3. Identification and Quantification of Volatile Compounds

The analysis of volatile compounds in the wine samples was conducted using the SPME-Arrow-GC/MS (gas chromatography–mass spectrometry) [15]. The SPME-Arrow extraction was performed using the RSH Triplus autosampler (Thermo Fisher Scientific Inc., Brookfield, WI, USA). A total of 5 mL of sample and 2.00 g of NaCl were put in 20 mL headspace screw-top vials sealed with PTFE/silicone septum-containing caps. The sorption conditions were as follows: the sample was incubated at 60 °C for 20 min and then SPME-Arrow fiber DVB/CWR/PDMS (120 µm × 20 mm; Thermo Fisher Scientific Inc., Brookfield, WI, USA) was exposed for 49 min. Then, the fiber was inserted into the GC injector port operating in splitless mode and was desorbed at 250 °C for 10 min.

Sample analysis was conducted on a TRACE 1300 Gas Chromatographer coupled to an ISQ 7000 TriPlus quadrupole mass spectrometer (Thermo Fisher Scientific Inc., USA) equipped with a TG-WAXMS A capillary column (60 m × 0.25 mm × 0.25 µm film thickness; Thermo Fisher Scientific, USA). The volatile compounds injected into the inlet were delivered to the column at a splitless mode and helium was used as a carrier gas at a constant flow rate of 1 mL/min. The oven temperature program was as follows: an initial temperature of 40 °C was maintained for 5 min, followed by an increase of 2 °C/min to 210 °C and being held for 10 min. MS spectra were recorded in the electron impact ionization mode (EI) at an ionization energy of 70 eV. Mass spectrometry was performed in full scan mode in the range of 30–300 *m*/*z*. The data obtained were processed using the Chromeleon Data System (Thermo Fisher Scientific Inc., USA). Identification was carried out by comparing retention times, retention index, and mass spectra with those of standards and with the data available in the Wiley Registry 12th Edition/NIST Spectral Library. Quantification was carried out using calibration curves. The curves (based on quantification ions) were constructed with Chromeleon 7 Chromatography Data System (CDS) software (version 7.2.10). As an internal standard, 3-methyl-3-pentanol was used at a final concentration of 1 mg/L. For all available standards, six different concentrations were prepared, while for the other compounds, semi-quantitative analysis was performed. Their concentrations were expressed in equivalents of similar compounds, with the assumption that a response factor was equal to one. The parameters of the identification and the calibration of wine volatiles are presented in Supplemental Table S1.

### 2.4. Determination of Organic Acids

Organic acids (tartaric, malic, lactic, citric, and succinic) were analyzed using High-performance Liquid Chromatography, Agilent 1050 (Palo Alto, CA, USA). The sample was previously filtered using PTFE membrane filters (0.45 μm). Identification and quantification were conducted at a wavelength of λ = 210 nm on Aminex HPX-87H (BioRad, Hercules, CA, USA).

### 2.5. Physicochemical Analysis

The basic physicochemical parameters were analyzed in must (reducing sugars) and wines (alcohol, total dry extract, total acidity, volatile acidity, pH, and ash) according to methods set by the International Organization of Vine and Wine (OIV, 2021) [16].

### 2.6. Sensory Analysis

For the 2020 vintage wines assessment, a panel of seven expert tasters participated (four females and three males), who were members of the Committee for Organoleptic Evaluation of Wine and Fruit Wines appointed by the Ministry of Agriculture. The panelists were specialists in the field and were well-experienced based on the evaluations in the Croatian Agency for Agriculture and Food, accredited according to the HRN EN ISO/IEC 17065 standard [17] for the implementation of the procedure for placing wines with PDO, i.e., certification of wines with a label of origin, on the market. Evaluation was performed in the Laboratory for Sensory Analysis of Agricultural and Food Products, University of Zagreb Faculty of Agriculture, in individual booths under standardized conditions.

**Figure 1:**
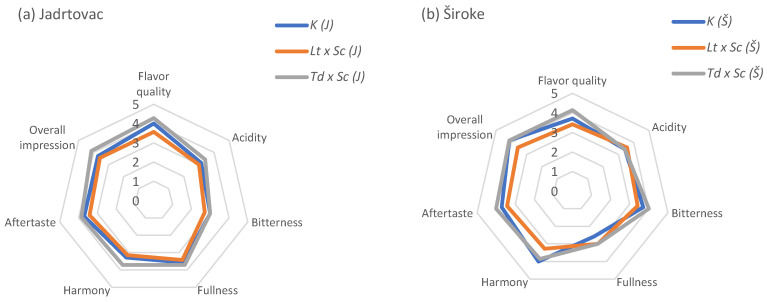
Quantitative descriptive sensory analysis of the taste parameters, cv. Babić red wine 2020, (**a**) Jadrtovac, (**b**) Široke.

**Figure 2:**
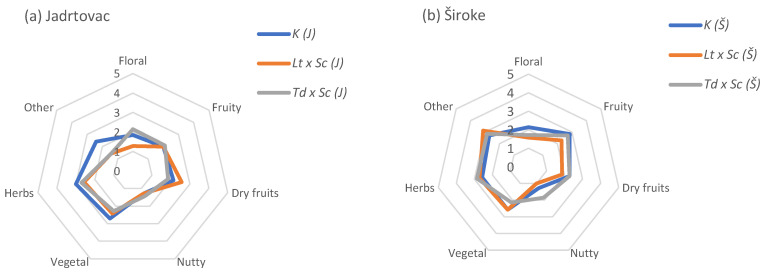
Quantitative descriptive sensory analysis of the odor parameters, cv. Babić red wine 2020, (**a**) Jadrtovac, (**b**) Široke.

All wines included in this study underwent sensory analysis using Quantitative Descriptive Analysis (QDA) six months after the conclusion of alcoholic fermentation. The Babić red wines (20 mL) were served at 15 °C in standard wine tasting glasses (ISO 3591:1977) [18] covered with a watch-glass to reduce the volatility of wine aromas. Blind tasting of coded samples was performed using three random replicates in the experiment. A total of 14 wine attributes for taste and odor (Figure 1 and Figure 2) were selected by the research group and were further developed and evaluated by panelists. The panel evaluated five referent monovarietal *cv*. Babić 2020 wines to achieve a consensus about the attributes describing wine’s sensory profiles. The additional training of the panel before the formal evaluation included assessing wine aroma using the aqueous solutions of different selected compounds and an “Aromaster” kit (Vinofil Co., Ltd., Hong Kong) that includes 88 typical wine aromas in vials (Supplemental Table S2). Quantification was performed using a six-point scale, on a paper sheet, as follows: 0–1—weak, 2–3—medium, and 4–5—strongly intensive attribute. Sample differences were graphically presented using radar graphs. Subsequently, the samples were ranked based on the overall quality, with the highest-ranked wine deemed the best, and the lowest-ranked wine identified as the worst.

### 2.7. Statistical Analysis

In the study of *cv*. Babić red wines, preconditions for applying ANOVA were examined using the Kolmogorov–Smirnov test for normality and the Bartlett test for variance homogeneity. All dependent variables met the conditions of normality and variance homogeneity, so a one-way analysis of variance (ANOVA) was applied. Differences in the chemical composition of wine (aromatic and phenolic compounds) between treatments and vineyard locations were tested using a two-way analysis of variance (ANOVA) with the SAS 9.4 statistical program (Cary, NC, USA).

## 3. Results and Discussion

### 3.1. Physicochemical Parameters

The study of the impact of non-*Saccharomyces* yeasts on the physicochemical parameters of *cv*. Babić wines pointed out significant differences among the treatments (Table 3). Based on the presented results, it can be seen that the prevalence of non-*Sc* yeasts at the start of fermentation impacted the overall composition of wine, which is consistent with prior published research [19]. Compared to the control, a lower alcohol content was determined in wines produced using *Lt x Sc* from the Jadrtovac location. The lower alcohol content can be attributed to the synthesis of lactic acid from sugars within the *Lt* metabolism [20], as confirmed by the results of organic acid analysis (Table 4). Depending on the chosen yeast strain and the conditions of alcoholic fermentation, the alcohol content of wine can be reduced by 1–2% (*v*/*v*) [21,22].

The total dry extract concentrations in wines were significantly the highest in the *Lt x Sc* treatments. The content of extract, resulting from the action of non-*Saccharomyces* yeasts, has a positive influence on taste properties [23].

Reducing sugar concentrations ranging from 3.40 g/L to 4.00 g/L, obtained through sequential fermentation, indicated the production of dry wines in both non-*Sc* treatments. Significantly, the lowest reducing sugar concentration was in the *Td x Sc* treatments. A higher reducing sugar concentration is attributed to a greater consumption of nutrients by *Lt* yeasts [24]. The addition of *Lt* yeasts to the must several days before the addition of *Sc* yeasts resulted in a depletion of nutrients for further activity.

This research revealed certain differences in the total acidity. Control treatments had lower concentrations of total acidity compared to the sequential fermentations, where it was higher for 1.20 in the *Lt x Sc* treatment and 0.50 g/L in the *Td x Sc* treatment. Earlier conducted studies [25,26,27] also pointed out a significant increase in total acidity in the sequential inoculation using *Sc x Lt* and *Sc x Td*. In the warmer climate of southeastern Europe, sequential inoculation can increase total acidity by up to 3.00 g/L [28]. Berbegal et al. stated that the maturity of grapes in warmer regions affects the concentration of total acidity, with tartaric acid being more stable at higher temperatures [29]. The use of *Lt* leads to the synthesis of lactic acid, which is an alternative to traditional malolactic fermentation in the production of red wine [30].

Recent research comparing several strains of *Lt* observed a significant degree of variability in volatile acidity, approximately 50% [31]. In this study, the range was 0.30–0.50 g/L with the highest concentrations in the *Td x Sc* (J) treatment. Comparing different strains of non-*Saccharomyces* yeasts [32] and three *Saccharomyces* yeasts, the reported concentrations were between 0.32 and 0.58 g/L for *Lt*, and 0.37 and 0.63 g/L for *Td* [29].

The pH values in the study ranged from 3.36 to 3.62, with significantly lower concentrations in sequential fermentations in the Jadrtovac locality, which was in line with the highest total acidity concentrations. Lower pH values were observed in all non-***Sc*** treatments. It was noted by Porter et al. (2019) [33] that the main reason for the reduction in pH values with the sequential fermentation process using *Lt* yeasts, by as much as 0.5 units at the beginning of fermentation, is related to lactic acid synthesis. According to Morata et al. (2018) [7], lower pH values at lower concentrations of total SO_2_ result in increased levels of molecular SO_2_, protecting against the effects of yeasts and bacteria such as *Brettanomyces* during aging. The same author noted positive effects in warmer climates affected by climate change, where the pH of the wine naturally decreases during fermentation without acid correction.

### 3.2. Organic Acids in Wines

Table 4 presents the results of the organic acids analysis. Significant differences were observed in the organic acids between treatments. It is well known that Babić wines have higher concentrations of tartaric acid in comparison to the other red Dalmatian wines. Sequential fermentations resulted in the same or significantly higher tartaric acid concentrations in the *Td x Sc* treatment. The increase in tartaric acid in sequential fermentation is yeast-dependent [34]. Given that tartaric acid is stable and only slightly variable, it is essential to maintain its stability, as noted before [35]. Sequential fermentation resulted in lower concentrations of malic acid at both locations. This is consistent with previous data [21] presenting a decrease in wines produced using the *Td* strain in comparison to the *Sc*.

There were significant differences in the concentrations of lactic acid, with the highest concentrations in the *Lt x Sc* treatments. *Lt* is known for the natural acidification of wine through the synthesis of lactic acid, but its action is effective only with co-inoculation with *Sc* [36,37]. One of the main criteria for selecting *Lt* is its ability to produce lactic acid, with a maximum concentration of 9.60 g/L [38], which exceeds the values obtained in this study. The synthesis of lactic acid influenced the total acidity of the wine, resulting in a significant increase in the *Lt x Sc* (J) treatment, which is in accordance with previously published data [7,31].

Wines also showed significant variation in the concentration of citric acid, and sequential fermentations influenced the reduction. The highest concentration was observed in the K(J), while the lowest was in the *Td x Sc* (J) treatment. Significant differences were also noted in the concentration of succinic acid, with higher concentrations in sequential fermentation treatments (J). Succinic acid is formed only during alcoholic fermentation influenced by various factors such as yeast strain, temperature, nitrogen content, and vitamins. In this case, the fermentation temperatures were consistent across all fermentations, while the yeasts varied, and there may have been differences in the amino acid profile and nitrogen content in the musts, as evidenced by significant variations in succinic acid concentrations at both locations. *Lt x Sc* (J) exhibited the highest succinic acid concentration, and a positive correlation between succinic acid and total acidity was observed. The role of succinic acid in elevating the total acidity of wine is also highlighted [39], which aligns with the findings of this study.

### 3.3. Volatile Aromatic Compounds in Wines

The results of the analysis of 101 volatile aroma compounds in Babić wines are shown in Table 5. There were no significant differences between all treatments regarding the total fatty acids, which ranged from 1044.00 μg/L to 1773.00 μg/L. The highest concentration was in the *Td x Sc* (J) treatment. Identical reductions during sequential fermentation with *Td* yeast are reported [40], while Belda et al. (2017) reported the unchanged concentration of fatty acids in wine produced by sequential fermentation [41]. The obtained results are consistent with those previously reported [42], and the reduction could result from the formation of smaller amounts of acetate and ethyl esters. A significant difference was observed only for hexanoic acid. Lower concentrations produced by sequential inoculation with Td yeast have been reported earlier [43]. A reduction in the concentrations of medium-chain fatty acids can be considered positive because of their contribution to negative aromas resembling fat, cheese, and even rancidity if present in higher concentrations [44]. Applied treatments did not significantly affect the concentration of total terpenes, which ranged from 122.49 μg/L K(J) to 159.85 μg/L (*Td x Sc* (Š)). The obtained results are in contradiction with a paper reporting the increase in total terpenes in other red grape varieties produced using *Lt x Sc* sequential inoculation [45]. Higher concentrations of linalool in wines produced by sequential fermentation with *Lt* and *Td* [45] were also not confirmed in this study. Significantly higher concentrations of geraniol were observed in treatments *Lt x Sc* and *Td x Sc* (J), which agrees with the influence of sequential inoculation with *Td* [45]. Nerol significantly differed in wines *Lt x Sc* (J) and *Td x Sc* (Š), similar to a previous study [46]. An increase in nerol and hotrienol was detected in sequential fermentation under the influence of *Lt* [29]. Certain strains of Td release conjugated terpenes that characterize specific wine varieties [45]. There is a scientific consensus regarding the positive influence of Td on the aromatic profile of the wine, which is also associated with the release of mannoproteins and the emphasizing of varietal characteristics [40]. A possible reason for this increase is closely related to the activity of glucosidase enzymes [30].

The concentration of total C_13_-norisoprenoids ranged from 1.66 μg/L to 3.97 μg/L. A significant difference was determined in the *Lt x Sc* and *Td x Sc* (J) treatments. *β*-damascenone and *β*-ionone are carriers of floral and fruity aromas [32]. β-damascenone significantly differed in the *Lt x Sc* and *Td x Sc* (J) treatments. As reported before [47], a significant increase in *β*-damascenone is noticed when Lt and Td yeast are used compared to *Sc* yeast. According to Korenika et al. (2021) [46], *Lt* did not affect the total concentration of C_13_-norisoprenoids regardless of the variety. In wines from both locations, TDN was not detected.

Total higher alcohols concentrations ranged from 36,311.00 (K(J)) to 42,080.00 μg/L (*Lt x Sc* (J)), with no significant differences between the treatments. Studies report a decrease in total higher alcohols produced during sequential inoculation with *Lt* yeast [25,28,48]. The *Lt x Sc* (Š) treatment had lower concentrations of total higher alcohols, although without statistical significance. This decrease could be due to strain variability within the *Lt* species and oxygen availability [49,50]. Wines produced using conventional *Sc* yeast have the highest concentrations of isoamyl alcohol compared to the *Lt* yeast [51]. All wines in this study have exceeded the isoamyl alcohol detection threshold of 300.00 mg/L [32,52]. Isoamyl alcohol has a strong sensory effect on wine [53], and sequential inoculation with *Lt* reduces the concentration of isoamyl alcohol in Sangiovese wines compared to *Sc* [25,48]. A significant difference was observed for 1-decanol at both localities.

Esters are volatile compounds produced by yeasts during alcoholic fermentation and contribute to the fruity aroma of wine. The intensity of fruitiness is mainly related to higher concentrations of esters and lower concentrations of alcohols and fatty acids [54]. Total esters in this study have shown significant differences at both localities. The *Lt x Sc* (J) treatment showed the highest concentration. Non-*Sc* yeasts are known to increase ester concentrations, but some studies report a decrease in certain ethyl esters compared to *Sc* [55]. Commercial *Sc* strains are known to produce esters such as isoamyl acetate, hexyl acetate, ethyl hexanoate, and ethyl octanoate, which affect the aromatic profile of wine [32]. Sequential inoculation with non-*Sc* yeasts is one way to increase acetate ester concentration [56]. There was a significant change in 2-phenylethyl acetate concentration in the *Lt x Sc* (J) treatment. A significant increase in ethyl lactate was observed in wines produced using *Lt* yeast and sequential fermentation with *Lt* yeast [47,53]. This corresponds to the highest concentration of ethyl lactate found in the *Lt x Sc* (J) treatment.

Aldehydes are volatile aromatic compounds that can be produced by non-*Sc* yeasts during alcoholic fermentation [4]. Depending on their thickness, certain aldehydes, esters, and terpenes can be adsorbed onto yeast cell walls, leading to a decrease in their concentration [57]. Total aldehydes ranged from 25.33 μg/L (*Td x Sc* (J)) to 39.15 μg/L (*Lt x Sc* (Š)) without a significant difference between treatments. A significant increase in 2-octenal in Babić and Trnjak wines produced throughout sequential inoculation with *Lt* yeasts contradicts the results of this study [46].

Lactones were found in overripe Syrah grapes, and their presence was confirmed in the Riesling variety, contributing to varietal aroma [58,59]. Most individual lactones have a positive effect on the wine aroma [60]. An increase in their concentration is attributed to the dominance of *Td* over *Sc*, resulting in a reduction in the amount of common ethyl esters [61]. This reduction in esters simultaneously leads to an increase in lactone concentration, achieving a better sensory effect on white wines [35]. The *Lt x Sc* (J) treatment resulted in the highest concentration of total lactones (590 μg/L), while the lowest concentration of 251 μg/L was determined in (K (J)). The most represented lactones in wine are butyrolactone and γ-butyrolactone [62]. This study found a significant increase in γ-butyrolactone in *Lt x Sc* (J), which is consistent with previous reports [46,51]. *Td x Sc* (J) wine had significantly higher concentrations of γ-nonalactone and δ-decalactone, which is in accordance with work by Azzolini et al. (2012) [63]. γ-decalactone dominated in wines from *Lt x Sc* treatments on both positions, similar to the study of white wines from warm regions [64].

Volatile phenols are classified as aromatic compounds, and the most significant representatives are vinyl and ethyl derivatives. Especially noteworthy is 4-ethylphenol, responsible for unpleasant odors such as ‘horse sweat or ‘barnyard’. Concentrations above the sensitivity threshold (0.23 mg/L) have a negative effect on the wine aroma [31]. *Sc* yeasts produce very low concentrations of volatile phenols during alcoholic fermentation due to their low level of hydroxycinnamic acid decarboxylation [65]. This was confirmed by the results for volatile phenol analysis in K (J) (99.50 μg/L) in this study. A possible reason for the lower concentration of volatile phenols is the earlier inoculation of non-*Saccharomyces* yeasts, which blocks the action of decarboxylase enzymes [31]. A significant difference between the control treatment with *Sc* and wines from sequential inoculation treatments with *Lt* and *Td* was observed [66]. Treatments *Td x Sc* resulted in the highest concentrations of total volatile phenols, which is in accordance with the study on Trnjak red wine [46].

During this research, a significant increase in the other compounds was observed in wines from sequential inoculations with *Lt* and *Td*. The only compound that significantly differed was 6-methyl-5-hepten-2-one in the *Lt x Sc* (J) treatment, which is not in accordance with a previous study [51].

### 3.4. Sensory Analysis

The results of a quantitative descriptive sensory analysis of the aroma and flavor properties of Babić wines from the Jadrtovac and Široke localities are shown in Figure 1 and Figure 2. Sequential fermentation with different yeast strains contributes to the enhanced wine flavor. This was achieved through the formation of various volatile compounds, including fatty acids, alcohols, esters, phenols, terpenes, and C_13_-norisoprenoids. It can be seen that *Td x Sc* wines were the best ranked in terms of flavor quality, fullness or body, aftertaste, and overall impression for both localities (Figure 1). *Lt x Sc* treatments had the lowest intensity of all taste parameters for both localities, except for the acidity in (Š). The obtained results were in accordance to the ranking method results, where the best-rated Babić red wine was *Td x Sc* (Š), and the lowest ranked wines on both positions were those from *Lt x Sc* treatments.

More pronounced differences in sensory properties between the treatments and positions were presented in odor parameters evaluation (Figure 2). Different treatments resulted in different aroma descriptions regarding the position. The best-ranked wine according to the overall impression, in this study—*Td x Sc* (Š) —showed a stronger intensity of fruity, dry fruits, nutty, and herbal aromas, and the lowest vegetal odors intensity (Figure 2b). More intense fruity aromas in these wines were associated with higher concentrations of ethyl hexanoate, which gives aromas of green apple, orange juice, and grapefruit, detected in the *Td x Sc* (Š) treatment. Additionally, the highest concentrations of ethyl 2-hydroxy-3-methylbutanoate contributed to the fruitiness. *Td x Sc* (Š) wine also had a significantly higher concentration of ethyl butanoate, which emits aromas resembling pineapple and apple. Moreover, the higher concentrations of ethyl hexanoate likely influenced the fruity odor, as the concentrations exceeded the sensory threshold of 0.014 mg/L [60]. *Td x Sc* (Š) treatment demonstrated a significant effect on the terpene composition, particularly on ethyl 3-hydroxybutanoate, which emits fruity and grape scents. The intensity of the rose-like floral scent was similar in the *Lt x Sc* (J) and *Td x Sc* (J) treatments. *Td x Sc* (J) wine exhibited the highest concentration of nerol, which resembles a rose and thyme odor. This study revealed a significant increase in *β*-damascenone in *Lt x Sc* (J) and *Td x Sc* (J), which emits fruity scents, particularly plum and honey [65].

The *Lt x Sc* (J) treatment exhibited the highest concentrations of ethyl lactate. Sequential fermentation with *Lt* strains significantly reduced the concentration of diethyl succinate, which emits scents reminiscent of ripe and overripe fruit. Wines from the *Lt x Sc* (J) treatment showed a significant decrease in *β*-ionone-5,6-epoxide. This treatment also displayed a significant decrease in nerol concentration, which resembles lemon-like aromas. *Lt x Sc* (J) treatment showed a significant increase in 3-ethoxy-1-propanol, which resembles aromas of blackcurrant and green pepper [67]. The concentration in this treatment was 15 folds higher than in (K), which could have a positive influence. Among other odor descriptors in *Lt x Sc* (Š) wines, the presence of cheese and buttery notes were detected probably due to higher concentrations of some fatty acids.

## 4. Conclusions

Based on the presented research, it can be concluded that sequential fermentation using non-*Saccharomyces* yeast species, specifically *Torulaspora delbrueckii x Saccharomyces cerevisiae* (*Td x Sc*) and *Lachancea thermotolerans x Saccharomyces cerevisiae* (*Lt x Sc*), had a significant impact on the chemical composition and sensory properties of cv. Babić red wines from two different vine-growing positions. The sequential fermentation partially resulted in a significant reduction in alcoholic strength and an increase in total acidity. Furthermore, a significant increase in tartaric, citric, and succinic acids was reported. In the *Lt x Sc* treatment, lactic acid synthesis occurred, as expected. *Td x Sc* (J) and *Lt x Sc* (Š) led to an increase in total esters and C_13_-norisoprenoids concentrations, as well as a significant decrease in total aldehydes, fatty acids, higher alcohols, and volatile phenols.

The *Td x Sc* treatments exhibited particularly positive aromatic properties, together with more intense fullness, harmony, aftertaste, and overall impression, while a more acidic taste was pronounced in the *Lt x Sc* treatment. However, the sensory properties of the *Lt x Sc* Babić red wines were negatively impacted, resulting in the lowest-rated wines regardless of positions. Further research is required to confirm the effects of non-*Sc* yeasts on the chemical composition and sensory properties of warm-climate red wines from other grape varieties and on large-scale production.

## Figures and Tables

**Table 1:** Inoculums for each treatment in the present study.

*K*	*Lt x Sc*	*Td x Sc*
Control treatment, alcoholic fermentation with the yeast strain *Saccharomyces cerevisiae* (Uvaferm BDX^®^, Lallemand Montreal, QC, Canada)	Sequential alcoholic fermentation with the yeast strains *Lachancea thermotolerans* (Laktia^®^, Lallemand, Montreal, QC, Canada) + *Saccharomyces cerevisiae* (Uvaferm BDX^®^, Lallemand, Montreal, QC, Canada)	Sequential alcoholic fermentation with the yeast strains *Torulaspora delbrueckii* (Biodiva^®^, Lallemand, Montreal, QC, Canada) + *Saccharomyces cerevisiae* (Lalvin ICV D254^®^, Lallemand, Montreal, QC, Canada)

**Table 2:** Protocols for the treatments.

*K*	*Lt x Sc*	*Td x Sc*
- Potassium metabisulfite (K_2_S_2_O_5_)—10 g/hL - pectolytic enzyme Lallzyme^®^ OE (2 g/hL, Lallemand, Montreal, QC, Canada) - inactive yeast Go-Ferm Protect^®^ (20 g/hL, Lallemand, Montreal, QC, Canada) - *Saccharomyces cerevisiae* Uvaferm BDX^®^ (25 g/hL, Lallemand, Montreal, QC, Canada) - yeast nutrient Fermaid E^®^ (20 g/hL, Lallemand, Montreal, QC, Canada)	- Potassium metabisulfite (K_2_S_2_O_5_)—3 g/hL - pectolytic enzyme Lallzyme^®^ OE (2 g/hL, Lallemand, Montreal, QC, Canada) - *Lachancea thermotolerans* Laktia^®^ (25 g/hL, Lallemand, Montreal, QC, Canada) + after 72 h, *Saccharomyces cerevisiae* Uvaferm BDX^®^ (25 g/hL, Lallemand, Montreal, QC, Canada) - yeast nutrient Fermaid E^®^ (20 g/hL, Lallemand, Montreal, QC, Canada)	- Potassium metabisulfite (K_2_S_2_O_5_)—3 g/hL - pectolytic enzyme Lallzyme^®^ OE (2 g/hL, Lallemand, Montreal, QC, Canada) - *Torulaspora delbrueckii* Biodiva^®^ (25 g/hL, Lallemand, Montreal, QC, Canada) + after 72 h, *Saccharomyces cerevisiae* Lalvin ICV D254^®^ (25 g/hL, Lallemand, Montreal, QC, Canada) - yeast nutrient Fermaid E^®^ (20 g/hL, Lallemand, Montreal, QC, Canada)

**Table 3:** Physicochemical parameters of Babić red wines, vintage 2020.

Compound	Location	Treatment		
		*K*	*Lt x Sc*	*Td x Sc*
Alcohol (vol%)	J	13.60 ± 0.01 c	13.30 ± 0.06 b	13.40 ± 0.02 b
	Š	13.00 ± 0.02 a	13.01 ± 0.03 a	12.93 ± 0.05 a
Total dry extract (g/L)	J	35.40 ± 0.20 c	37.50 ± 0.40 d	35.20 ± 0.20 bc
	Š	34.60 ± 0.20 ab	36.20 ± 0.20 d	34.40 ± 0.20 a
Reducing sugars (g/L)	J	3.80 ± 0.20 abc	4.00 ± 0.10 bc	3.40 ± 0.20 a
	Š	4.20 ± 0.10 c	3.70 ± 0.10 ab	3.40 ± 0.20 a
Total acidity * (g/L)	J	6.30 ± 0.10 a	7.50 ± 0.10 b	6.80 ± 0.20 ab
	Š	7.50 ± 0.10 b	8.20 ± 0.10 c	8.00 ± 0.20 c
Volatile acidity ** (g/L)	J	0.30 ± 0.02 ab	0.40 ± 0.01 ab	0.50 ± 0.01 a
	Š	0.42 ± 0.03 c	0.44 ± 0.04 c	0.40 ± 0.01 c
pH	J	3.62 ± 0.02 b	3.51 ± 0.01 a	3.53 ± 0.02 a
	Š	3.36 ± 0.01 a	3.32 ± 0.02 a	3.32 ± 0.02 a
Ash (g/L)	J	3.35 ± 0.01 a	3.48 ± 0.01 b	3.40 ± 0.10 ab
	Š	3.29 ± 0.01 a	3.39 ± 0.01 ab	3.35 ± 0.02 a
Total phenols (mg/L)	J	1775.00 ± 300.52 a	1632.50 ± 38.89 a	1610.00 ± 162.63 a
	Š	1567.50 ± 24.74 b	1590.00 ± 134.35 b	1520.00 ± 14.14 b

* tartaric acid and ** acetic acid equivalents. Concentrations are expressed as mean ± standard deviation (*n* = 3). Different letters in the rows represent statistically significant differences between treatments at the significance level of *p* < 0.05, separately for two localities (two-way ANOVA and LSD test). Different letters in the columns represent statistically significant differences between localities of the same treatment at the significance level of *p* < 0.05. J—Jadrtovac, Š—Široke, K—control treatment (*S. cerevisiae*), *Lt x Sc*—*L. thermotolerans x S. cerevisiae*, *Td x Sc*—*T. delbrueckii x S. cerevisiae*.

**Table 4:** Concentration of organic acids (g/L).

Organic Acid (g/L)	Location	Treatment		
		*K*	*Lt x Sc*	*Td x Sc*
Tartaric	J	2.76 ± 0.01 a	2.83 ± 0.02 b	2.96 ± 0.02 c
	Š	4.12 ± 0.04 d	4.18 ± 0.02 d	4.38 ± 0.02 e
Malic	J	0.29 ± 0.00 d	0.26 ± 0.00 c	0.27 ± 0.00 c
	Š	0.18 ± 0.01 b	0.17 ± 0.01 b	0.15 ± 0.01 a
Lactic	J	0.01 ± 0.01 a	1.58 ± 0.01 d	0.06 ± 0.01 b
	Š	0.01 ± 0.01 a	0.62 ± 0.01 c	0.06 ± 0.01 b
Citric	J	0.49 ± 0.01 c	0.35 ± 0.01 b	0.26 ± 0.00 a
	Š	0.33 ± 0.01 b	0.34 ± 0.02 b	0.27 ± 0.01 a
Succinic	J	0.43 ± 0.00 a	0.71 ± 0.02 d	0.58 ± 0.01 c
	Š	0.54 ± 0.01 b	0.42 ± 0.02 a	0.42 ± 0.01 a

Concentrations are expressed as mean ± standard deviation (*n* = 3). Different letters in the rows represent statistically significant differences between treatments at the significance level of *p* < 0.05, separately for two localities (two-way ANOVA and LSD test). Different letters in the columns represent statistically significant differences between localities of the same treatment at the significance level of *p* < 0.05. J—Jadrtovac, Š—Široke, K—control treatment (*S. cerevisiae*), *Lt x Sc*—*L. thermotolerans x S. cerevisiae*, *Td x Sc*—*T. delbrueckii x S. cerevisiae*.

**Table 5:** Concentrations of volatile aroma compounds (μg/L) in *cv*. Babić red wines.

Compounds (μg/L)	Locality	Treatments		
		*K*	*Lt x Sc*	*Td x Sc*
Fatty acids				
Propanoic acid	J	3.68 ± 0.28 a	2.86 ± 0.26 a	2.60 ± 0.00 a
	Š	2.70 ± 0.12 a	2.70 ± 0.12 a	3.20 ± 0.93 a
2-Methylpropanoic acid	J	521.71 ± 17.41 a	933.11 ± 558.58 a	1052.60 ± 50.55 a
	Š	535.41 ± 0.79 a	661.98 ± 19.50 a	762.14 ± 86.67 a
Butanoic acid	J	159.07 ± 2.39 a	191.02 ± 26.57 a	264.78 ± 13.21 a
	Š	217.19 ± 4.58 a	209.52 ± 14.63 a	153.61 ± 154.60 a
Isovaleric acid	J	2.63 ± 0.02 a	2.84 ± 0.13 a	2.69 ± 0.09 a
	Š	2.74 ± 0.01 a	2.79 ± 0.16 a	3.29 ± 0.43 a
Hexanoic acid	J	338.12 ± 20.36 a	309.98 ± 43.79 a	431.63 ± 6.05 ab
	Š	599.29 ± 71.17 b	467.86 ± 62.50 ab	543.92 ± 83.35 ab
Heptanoic acid	J	5.35 ± 2.43 a	8.03 ± 0.77 a	5.65 ± 2.92 a
	Š	9.03 ± 0.36 a	8.92 ± 0.68 a	6.02 ± 3.46 a
Nonanoic acid	J	7.95 ± 0.09 a	8.34 ± 0.21 a	8.20 ± 0.15 a
	Š	8.26 ± 0.09 a	8.11 ± 0.38 a	8.43 ± 0.21 a
Decanoic acid	J	5.46 ± 0.47 a	5.01 ± 1.56 a	5.17 ± 0.01 a
	Š	5.04 ± 0.22 a	4.76 ± 0.41 a	5.00 ± 0.19 a
Ʃ Fatty acids	J	1044.00 ± 37.10 a	1461.00 ± 489.00 a	1773.00 ± 60.70 a
	Š	1380.00 ± 76.70 a	1367.00 ± 97.90 a	1486.00 ± 12.50 a
Terpenes				
Farnesol	J	10.38 ± 4.85 a	8.90 ± 0.37 a	10.57 ± 0.88 a
	Š	6.27 ± 3.66 a	3.01 ± 0.22 a	7.12 ± 5.42 a
Tetrahydrolinalool	J	6.43 ± 0.33 a	11.07 ± 0.02 a	30.10 ± 0.08 b
	Š	12.42 ± 0.67 a	5.76 ± 8.15 a	12.32 ± 1.66 a
Linalyl format	J	0.36 ± 0.14 a	0.42 ± 0.00 a	0.32 ± 0.12
	Š	1.51 ± 0.61 a	1.29 ± 0.24 a	1.27 ± 0.17 a
*cis*-Linalool oxide, fur.	J	1.60 ± 0.19 a	2.19 ± 0.03 ab	1.59 ± 0.03 a
	Š	3.23 ± 0.24 bc	3.59 ± 0.14 c	3.98 ± 0.55 c
Linalool	J	4.48 ± 0.00 a	4.38 ± 0.74 a	4.43 ± 0.16 a
	Š	4.82 ± 0.85 a	4.39 ± 0.09 a	4.34 ± 0.62 a
Terpinen-4-ol	J	4.14 ± 1.30 a	7.81 ± 0.92 b	5.89 ± 0.22 ab
	Š	4.57 ± 0.09 a	5.58 ± 0.22 ab	4.44 ± 0.17 a
Hotrienol	J	0.59 ± 0.24 a	1.56 ± 1.01 ab	1.55 ± 1.32 ab
	Š	4.54 ± 2.44 a	3.34 ± 0.67 ab	3.12 ± 0.53 a
β-Ionone-5,6-epoxide	J	0.13 ± 0.02 a	0.10 ± 0.05 b	0.16 ± 0.04 ab
	Š	0.14 ± 0.04 a	0.09 ± 0.10 ab	0.15 ± 0.08 a
*cis-β*-Farnesene	J	0.56 ± 0.02 a	0.59 ± 0.00 a	0.56 ± 0.01 a
	Š	0.83 ± 0.22 a	0.71 ± 0.24 a	0.55 ± 0.02 a
*trans*-*β*-Farnesene	J	0.87 ± 0.04 a	1.05 ± 0.14 a	1.24 ± 0.02 a
	Š	0.90 ± 0.17 a	1.06 ± 0.07 a	0.90 ± 0.49 a
Menthol	J	0.29 ± 0.16 a	0.19 ± 0.24 a	0.12 ± 0.04 a
	Š	0.25 ± 0.17 a	0.25 ± 0.21 a	0.33 ± 0.04 a
Ocimenol	J	0.18 ± 0.20 a	0.20 ± 0.20 a	0.05 ± 0.01 a
	Š	0.40 ± 0.25 a	0.18 ± 0.06 a	0.36 ± 0.18 a
Nerolic acid	J	11.68 ± 1.50 b	2.48 ± 0.14 a	2.39 ± 0.38 a
	Š	2.53 ± 0.02 a	12.59 ± 0.90 b	12.95 ± 0.45 b
2,6-Dimethyl-3,7-octadien-2,6-diol	J	0.19 ± 0.02 a	0.33 ± 0.07 a	0.37 ± 0.00 a
	Š	0.47 ± 0.51 a	0.16 ± 0.02 a	0.32 ± 0.06 a
*α*-Terpineol	J	1.73 ± 0.51 a	2.23 ± 0.65 a	1.98 ± 0.19 a
	Š	1.75 ± 0.14 a	1.67 ± 0.00 a	2.14 ± 0.48 a
Terpendiol I	J	4.2 ± 0.64 b	4.15 ± 0.72 b	3.32 ± 0.07 ab
	Š	2.48 ± 0.04 ab	1.60 ± 0.12 a	1.86 ± 0.24 a
Citronellol	J	31.77 ± 3.91 a	28.02 ± 4.12 a	31.98 ± 0.60 a
	Š	27.83 ± 0.10 a	21.01 ± 0.51 a	32.52 ± 3.51 a
Nerol	J	1.02 ± 0.05 a	2.55 ± 0.14 b	3.26 ± 0.00 c
	Š	2.52 ± 0.09 b	2.66 ± 0.14 bc	2.35 ± 0.25 b
Geraniol	J	6.85 ± 0.74 a	10.58 ± 1.41 b	10.33 ± 0.09 b
	Š	5.57 ± 0.05 a	5.08 ± 0.09 a	4.50 ± 0.31 a
Terpendiol II	J	0.63 ± 0.77 a	0.16 ± 0.09 a	0.35 ± 0.31 a
	Š	1.14 ± 0.24 a	0.49 ± 0.23 a	0.93 ± 0.84 a
6,7-Dihydro-7-hydroxylinalool	J	10.11 ± 0.53 a	14.01± 0.50 a	13.71 ± 2.72 a
	Š	23.01 ± 0.50 a	20.56 ± 1.84 a	23.18 ± 6.07 a
2,6-Dimethyl-7-octen-2,6-diol	J	9.33 ± 1.65 a	12.59 ± 1.37 ab	12.11 ± 1.38 ab
	Š	21.38 ± 1.35 bc	19.29 ± 1.83 abc	22.86 ± 3.93 c
Nerolidol	J	0.59 ± 0.09 a	0.63 ± 0.03 a	0.89 ± 0.35 a
	Š	0.76 ± 0.02 a	0.76 ± 0.26 a	0.82 ± 0.07 a
1,8-Terpin	J	1.10 ± 0.96 a	1.66 ± 0.41 a	1.17 ± 1.15 a
	Š	1.45 ± 1.68 a	1.10 ± 1.15 a	1.32 ± 1.56 a
Geranyl acetate	J	9.84 ± 0.33 a	11.20 ± 0.06 a	11.79 ± 1.90 a
	Š	14.39 ± 1.34 a	11.69 ± 0.48 a	12.07 ± 1.65 a
8-Hydroksylinalool	J	1.86 ± 1.83 a	1.28 ± 1.18 a	2.15 ± 2.02 a
	Š	8.86 ± 11.59 a	0.89 ± 0.28 a	1.89 ± 1.40 a
Ethyl linalyl acetate	J	1.91 ± 1.21 a	0.33 ± 0.24 a	0.95 ± 0.45 a
	Š	0.87 ± 0.12 a	0.72 ± 0.04 a	1.06 ± 0.16 a
Ʃ Terpenes	J	122.49 ± 14.10 a	130.98 ± 9.56 a	153.68 ± 3.55 a
	Š	154.55 ± 0.84 a	129.82 ± 11.70 a	159.85 ± 27.60 a
C_13_-norisoprenoids				
*β*-Damascenone	J	2.23 ± 0.19 b	3.75 ± 0.28 c	3.25 ± 0.05 c
	Š	1.18 ± 0.12 a	1.65 ± 0.26 ab	1.53 ± 0.18 ab
TDN	J	n.d.	n.d.	n.d.
	Š	n.d.	n.d.	n.d.
*β*-Ionone	J	0.12 ± 0.04 a	0.13 ± 0.02 ab	0.12 ± 0.04 a
	Š	0.30 ± 0.02 b	0.20 ± 0.00 ab	0.27 ± 0.04 ab
*α*-Ionone	J	0.10 ± 0.14 a	0.08 ± 0.12 a	0.22 ± 0.00 a
	Š	0.18 ± 0.01 a	0.20 ± 0.04 a	0.17 ± 0.00 a
Ʃ C_13_-norisoprenoids	J	2.45 ± 0.38 a	3.97 ± 0.18 b	3.60 ± 0.00 b
	Š	1.66 ± 0.16 a	2.06 ± 0.21 a	1.98 ± 0.14 a
Higher alcohols				
Isobutanol	J	5130.80 ± 278.80 bc	5415.53 ± 71.36 ab	3941.29 ± 237.07 a
	Š	429.55 ± 467.41 abc	4206.45 ± 69.14 c	4322.40 ± 137.48 abc
1-Butanol	J	139.92 ± 16.40 a	167.33 ± 5.61 a	136.29 ± 2.85 a
	Š	140.27 ± 31.10 a	196.66 ± 4.29 a	170.33 ± 8.69 a
2-Methyl-1-butanol	J	11,114.39 ± 1069.30 a	21,785.33 ± 250.97 a	20,076.06 ± 473.04 a
	Š	20,415.29 ± 628.22 a	20,973.72 ± 51.15 a	21,340.38 ± 131.95 a
Isoamyl alcohol	J	12,793.67 ± 8.23 a	8048.26 ± 9.82 a	8419.33 ± 8.10 a
	Š	10,245.44 ± 3.73 a	8827.16 ± 1.13 a	8914.66 ± 5.97 a
4-Methyl-1-pentanol	J	37.455 ± 1.47 a	32.15 ± 6.20 a	26.20 ± 5.18 a
	Š	27.72 ± 0.75 a	26.13 ± 1.20 a	30.34 ± 5.21 a
1-Octanol	J	0.75 ± 0.94 a	1.00 ± 0.52 a	0.03 ± 0.00 a
	Š	0.13 ± 0.03 a	0.28 ± 0.31 a	0.33 ± 0.33 a
1-Nonanol	J	7.23 ± 1.12 ab	5.53 ± 1.29 ab	4.85 ± 0.07 a
	Š	7.49 ± 0.04 ab	6.06 ± 0.57 ab	8.70 ± 0.53 b
2-Penten-1-ol	J	8.66 ± 0.39 a	8.61 ± 1.34 a	6.46 ± 1.4 a
	Š	5.91 ± 0.48 a	6.01 ± 0.09 a	6.80 ± 1.35 a
1-Hexanol	J	1179.26 ± 47.80 a	1239.97 ± 203.88 a	1412.61 ± 27.33 a
	Š	1649.18 ± 57.89 a	1409.09 ± 30.79 a	1510.73 ± 195.77 a
*trans*-3-Hexen-1-ol	J	22.65 ± 2.11 a	24.76 ± 4.24 a	22.96 ± 0.19 a
	Š	42.88 ± 0.10 b	41.63 ± 0.94 b	43.26 ± 6.54 b
3-Etoxy-1-propanol	J	7.08 ± 1.35 a	121.48 ± 27.28 b	48.35 ± 2.80 a
	Š	15.13 ± 0.30 a	42.08 ± 2.51 a	7.67 ± 0.72 a
*cis*-3-Hexen-1-ol	J	12.49 ± 0.16 ab	15.15 ± 2.48 ab	10.46 ± 0.26 a
	Š	18.96 ± 0.16 ab	20.61 ± 0.28 b	20.50 ± 3.76 b
*trans*-3-Hexen-1-ol	J	4.83 ± 0.26 a	5.90 ± 0.14 a	3.77 ± 0.16 a
	Š	12.01 ± 0.26 b	11.39 ± 0.41 b	12.40 ± 1.38 b
2-Ethyl-1-hexanol	J	0.11 ± 0.08 a	0.14 ± 0.14 a	0.04 ± 0.00 a
	Š	0.41 ± 0.48 a	0.65 ± 0.07 a	0.25 ± 0.19 a
1-Decanol	J	3.83 ± 0.40 bc	2.10 ± 0.14 a	2.48 ± 0.06 ab
	Š	4.22 ± 0.23 c	2.31 ± 0.33 a	3.87 ± 0.48 bc
Phenylethyl alcohol	J	5847.45 ± 171.74 a	5207.13 ± 28.62 a	5302.40 ± 29.76 a
	Š	4634.85 ± 72.85 a	2768.87 ± 2452.29 a	2721.91 ± 1921.65 a
Ʃ Higher alcohols	J	36,311.00 ± 236.00 a	42,080.00 ± 592.00 a	39,414.00 ± 565.00 a
	Š	41,516.00 ± 623.00 a	38,539.00 ± 3504.00 a	39,163.00 ± 189.00 a
Esters				
Isobutyl acetate	J	80.59 ± 30.38 a	107.63 ± 0.47 a	64.89 ± 0.49 a
	Š	81.74 ± 1.02 a	81.30 ± 0.17 a	98.57 ± 3.09 a
Ethyl butanoate	J	113.9 ± 6.06 a	113.25 ± 4.53 a	159.915 ± 2.58 ab
	Š	172.53 ± 0.26 ab	176.33 ± 7.00 ab	202.57 ± 34.18 b
Isoamyl acetate	J	333.97 ± 38.13 a	514.63 ± 89.22 a	576.81 ± 38.76 a
	Š	607.65 ± 9.61 a	658.69 ± 40.51 a	688.53 ± 196.17 a
Ethyl hexanoate	J	68.59 ± 15.28 ab	59.06 ± 9.04 a	93.98 ± 4.96 ab
	Š	163.6 ± 0.85 c	128.17 ± 1.45 bc	173.26 ± 25.58 c
Ethyl lactate	J	185.44 ± 1.35 a	1826.19 ± 347.42 b	385.48 ± 27.91 a
	Š	282.66 ± 15.52 a	608.89 ± 8.36 b	402.41 ± 46.77 a
Ethyl 2-hydroxy-3-methylbutanoate	J	1.71 ± 0.04 a	3.45 ± 0.07 b	10.14 ± 0.03 c
	Š	3.88 ± 0.23 b	3.46 ± 0.14 b	3.92 ± 0.70 b
Ethyl octanoate	J	31.08 ± 1.67 ab	19.89 ± 1.35 a	27.32 ± 3.30 ab
	Š	72.50 ± 3.13 ab	47.96 ± 3.36 ab	80.78 ± 29.86 b
Ethyl 3-hydroxybutanoate	J	11.70 ± 0.63 a	11.57 ± 2.31 a	14.03 ± 0.82 a
	Š	18.42 ± 1.01 a	16.81 ± 0.14 a	31.02 ± 3.73 b
Ethyl furoate	J	2.24 ± 0.17 b	2.62 ± 0.31 bc	3.51 ± 0.04 c
	Š	0.28 ± 0.04 a	3.06 ± 0.02 bc	3.82 ± 0.54 c
Diethyl succinate	J	155.10 ± 7.17 b	73.13 ± 7.87 a	184.26 ± 3.63 b
	Š	167.58 ± 1.04 b	149.10 ± 2.34 b	191.31 ± 27.18 b
2-Phenylethyl acetate	J	1.10 ± 0.20 ab	1.86 ± 0.29 b	1.66 ± 0.19 ab
	Š	1.05 ± 0.02 ab	1.04 ± 0.01 a	0.94 ± 0.11 a
Diethyl malate	J	5.62 ± 0.05 a	5.62 ± 0.31 a	10.21 ± 0.82 ab
	Š	8.25 ± 0.50 ab	8.69 ± 0.02 ab	12.90 ± 3.22 b
Ethyl hydrogen succinate	J	0.22 ± 0.19 a	0.50 ± 0.07 a	0.83 ± 0.15 a
	Š	0.77 ± 0.20 a	0.71 ± 0.20 a	0.37 ± 0.07 a
Ethyl linoleate	J	0.16 ± 0.02 a	0.37 ± 0.05 a	0.21 ± 0.01 a
	Š	0.27 ± 0.00 a	0.00 ± 0.25 a	0.32 ± 0.20 a
Ethyl vanillate	J	5.30 ± 0.73 b	6.09 ± 0.26 bc	6.72 ± 0.24 bc
	Š	7.62 ± 0.08 c	7.16 ± 0.08 c	0.00 ± 0.00 a
Ʃ Esters	J	995.83 ± 25.90 a	2745.56 ± 273.00 c	1540.36 ± 20.60 ab
	Š	1588.58 ± 25.20 ab	1891.05 ± 31.40 bc	1890.31 ± 366.00 bc
Aldehydes				
2,4-Hexadienal	J	1.22 ± 0.00 a	1.29 ± 0.02 ab	1.27 ± 0.14 ab
	Š	1.56 ± 0.05 b	1.50 ± 0.02 ab	1.52 ± 0.06 ab
Benzaldehyde	J	12.62 ± 1.13 ab	10.18 ± 0.89 a	10.27 ± 0.09 a
	Š	16.15 ± 0.81 b	15.34 ± 1.17 b	12.11 ± 1.30 ab
2,4-Heptadienal (E)	J	8.14 ± 0.65 a	10.26 ± 1.18 a	9.62 ± 0.29 a
	Š	10.27 ± 0.39 a	9.10 ± 0.38 a	11.21 ± 1.61 a
Decanal	J	1.59 ± 0.18 a	2.53 ± 0.44 a	1.81 ± 0.12 a
	Š	3.22 ± 0.79 a	2.38 ± 0.17 a	2.71 ± 0.16 a
Acetylfuran	J	1.13 ± 0.09 a	1.25 ± 0.27 a	1.10 ± 0.07 a
	Š	1.18 ± 0.19 a	1.02 ± 0.12 a	1.43 ± 0.28 a
2,4-Nonadienal	J	1.33 ± 0.19 b	1.32 ± 0.20 b	1.04 ± 0.03 ab
	Š	0.79 ± 0.05 ab	0.52 ± 0.06 a	0.66 ± 0.06 a
2,4-Decadienal	J	0.17 ± 0.22 a	0.07 ± 0.09 a	0.21 ± 0.25 a
	Š	0.12 ± 0.03 a	9.02 ± 12.68 a	2.74 ± 1.24 a
2,4-Heptadienal (Z)	J	0.40 ± 0.00 a	0.11 ± 0.15 a	n.d.
	Š	0.28 ± 0.19 a	0.25 ± 0.00 a	0.35 ± 0.07 a
Ʃ Aldehydes	J	26.59 ± 0.45 a	27.06 ± 3.59 a	25.33 ± 1.30 a
	Š	33.60 ± 0.60 a	39.15 ± 14.6 a	32.72 ± 2.88 a
Lactones				
*γ*-Decalactone	J	1.27 ± 0.12 a	3.29 ± 0.03 bc	1.65 ± 0.24 ab
	Š	1.44 ± 0.33 a	3.96 ± 0.76 c	1.72 ± 0.28 ab
*γ*-Nonalactone	J	33.74 ± 2.25 a	31.44 ± 0.45 a	39.84 ± 5.61 a
	Š	35.64 ± 0.96 a	34.71 ± 0.89 a	49.18 ± 12.77 a
*γ*-Hexalactone	J	6.30 ± 0.28 a	6.82 ± 0.79 a	6.70 ± 0.37 a
	Š	8.38 ± 0.45 a	9.34 ± 0.04 ab	12.31 ± 1.62 b
*γ*-Octalactone	J	0.84 ± 0.07 b	1.93 ± 0.02 c	0.53 ± 0.03 ab
	Š	0.53 ± 0.07 ab	0.45 ± 0.01 ab	0.31 ± 0.20 a
*δ*-Decalactone	J	2.79 ± 0.15 a	2.71 ± 0.07 a	3.71 ± 0.16 b
	Š	2.71 ± 0.14 a	2.50 ± 0.11 a	2.89 ± 0.13 a
*γ*-Undecalactone	J	0.46 ± 0.04 ab	0.45 ± 0.07 ab	0.43 ± 0.02 a
	Š	0.41 ± 0.06 a	0.43 ± 0.01 a	0.60 ± 0.02 b
*γ*-Butyrolactone	J	325.83 ± 11.60 ab	543.00 ± 90.58 b	299.10 ± 35.63 a
	Š	201.61 ± 9.75 a	243.26 ± 5.72 a	327.91 ± 77.36 ab
Ʃ Lactones	J	371.00 ± 14.40 ab	590.00 ± 91.80 b	352.00 ± 30.00 ab
	Š	251.00 ± 9.45 a	295.00 ± 7.42 a	395.00 ± 91.80 ab
Volatile phenols				
Guaiacol	J	3.51 ± 0.19 b	3.10 ± 0.38 a	3.18 ± 0.00 b
	Š	1.32 ± 0.02 a	1.01 ± 0.01 a	1.48 ± 0.33 a
Homovanillyl alcohol	J	70.75 ± 1.93 a	75.03 ± 2.63 a	79.80 ± 0.96 a
	Š	115.85 ± 1.43 b	112.26 ± 2.77 b	132.01 ± 14.79 b
Eugenol	J	0.60 ± 0.12 ab	0.55 ± 0.04 ab	0.81 ± 0.015 b
	Š	0.30 ± 0.01 a	0.27 ± 0.03 a	0.36 ± 0.11 a
4-Ethylphenol	J	3.25 ± 0.04 b	1.90 ± 0.00 a	3.08 ± 0.14 b
	Š	2.53 ± 0.33 ab	2.52 ± 0.11 ab	3.40 ± 0.36 b
4-Vinylphenol	J	12.09 ± 1.32 a	9.76 ± 0.05 a	9.30 ± 0.73 a
	Š	7.30 ± 0.98 a	9.46 ± 1.35 a	7.60 ± 1.68 a
Vanillin	J	9.29 ± 2.74 a	12.13 ± 1.56 a	15.22 ± 1.48 a
	Š	15.28 ± 0.73 a	15.34 ± 0.48 a	17.58 ± 2.67 a
Ʃ Volatile phenols	J	99.50 ± 0.77 a	102.00 ± 1.47 ab	111.00 ± 1.70 b
	Š	143.00 ± 0.00 c	141.00 ± 2.06 c	162.00 ± 20.00 d
Other compounds				
2-Pentylfuran	J	245.51 ± 9.17 a	255.59 ± 4.92 a	264.88 ± 14.15 a
	Š	233.42 ± 2.08 a	248.72 ± 23.94 a	246.61 ± 23.49 a
Acetoin	J	12.84 ± 1.32 a	14.23 ± 6.73 a	8.69 ± 3.98 a
	Š	12.96 ± 0.79 a	15.82 ± 6.83 a	18.61 ± 4.90 a
6-Methyl-5-hepten-2-one	J	34.90 ±1.22 a	362.02 ± 67.38 b	73.19 ± 5.53 a
	Š	53.61 ± 4.17 a	117.57 ± 1.58 a	75.73 ± 8.88 a
Furfuryl alcohol	J	0.26 ± 0.24 a	0.47 ± 0.08 a	0.45 ± 0.25 a
	Š	3.78 ± 0.07 bc	3.02 ± 0.16 b	4.95 ± 0.53 c
4-Ethyl-cyclohexanol	J	8.75 ± 0.77 a	11.28 ± 1.40 a	10.51 ± 0.35 a
	Š	11.28 ± 0.46 a	9.89 ± 0.45 a	12.40 ± 1.90 a
Furfural	J	1.69 ± 0.37 a	2.08 ± 0.08 a	1.87 ± 0.67 a
	Š	1.96 ± 0.01 a	1.66 ± 0.31 a	1.33 ± 0.55 a
Benzyl alcohol	J	9.65 ± 0.38 a	8.36 ± 0.26 a	9.84 ± 0.74 a
	Š	7.82 ± 0.03 a	7.03 ± 0.00 a	8.79 ± 1.18 a
Ʃ Other compounds	J	314.00 ± 4.61 a	653.69 ±72.80 b	369.41 ± 8.25 a
	Š	327.57 ± 7.15 a	404.11 ± 19.00 a	368.83 ±38.70 a

Concentrations are expressed as mean ± standard deviation (*n* = 3). Different letters in the same row represent statistically significant differences between treatments at the significance level of *p* < 0.05, separately for each location (two-way ANOVA and LSD test). Different letters in the column represent statistically significant differences between locations of the same treatment at the significance level of *p* < 0.05. J—Jadrtovac, Š—Široke, *K*—control treatment (*S. cerevisiae*), *Lt x Sc*—*L. thermotolerans x S. cerevisiae*, *Td x Sc*—*T. delbrueckii x S. cerevisiae*, n.d.—not detected.

## Data Availability

The original contributions presented in the study are included in the article/Supplementary Material, further inquiries can be directed to the corresponding author.

## Supplementary Materials

The following supporting information can be downloaded at: https://www.mdpi.com/article/10.3390/foods13132000/s1, Supplemental Table S1. Parameters of the identification and calibration of wine volatile compounds. Supplemental Table S2. Sensory attributes and material used for the sensory panel training.
