# Supplementary material for: Sequential Fermentation in Red Wine cv. Babić Production: The Influence of Torulaspora delbrueckii and Lachancea thermotolerans Yeasts on the Aromatic and Sensory Profile
Source: Foods. 2024 Jun 25;13(13):2000. doi: 10.3390/foods13132000 (PMC11241832; doi:10.3390/foods13132000)
Supplement: Supplementary file 1 [file foods-13-02000-s001.zip › foods-3051206-Table S1.pdf]

Supplemental Table S1. Parameters of the identification and calibration of wine volatile compounds					
Group	Compound	RI (lit)	RI (det)	**Linear range (mg/L)**	**Determination coefficient (R^2)**
Acid	Isobutyric acid	1549	1541	50-5000	0.9934
Acid	Isovaleric acid	1667	1672	50-5000	0.9928
Acid	Benzoic acid	2380	2383	50-5000	0.9911
Acid	Butanoic acid	1624	1632	50-5000	0.9984
Acid	Decanoic acid	2274	2286	50-5000	0.9948
Acid	Dodecanoic acid	2487	2493	50-5000	0.9973
Acid	Hexanoic acid	1843	1849	50-5000	0.9966
Acid	Nonanoic acid	2159	2169	50-5000	0.9926
Acid	Octanoic acid	2057	2050	50-5000	0.9953
Acid	Undecanoic acid	2391	2388	50-5000	0.9941
Acid	Propanoic acid	1579	1588	50-5000	0.9938
Acid	2-Methylpropanoic acid	1604	1621	50-5000	0.9979
Acid	Heptanoic acid	1997	2005	50-5000	0.9968
Alcohol	1-Butanol	1140	1135	50-5000	0.9973
Alcohol	1-Decanol	1575	1568	50-5000	0.9952
Alcohol	2-Hexadecanol	2312	2305	50-5000	1.0000
Alcohol	1-Hexanol	1351	1359	50-5000	0.9948
Alcohol	1-Nonanol	1385	1388	50-5000	0.9958
Alcohol	1-Octanol	1392	1399	50-5000	0.9914
Alcohol	1-Pentanol	1410	1413	50-5000	0.9941
Alcohol	1-Propanol	1020	1025	50-5000	0.9972
Alcohol	2,3-Butanediol, (R,R)	1550	1548	50-5000	0.9971
Alcohol	2-Ethyl-1-hexanol	1990	1994	50-5000	0.9998
Alcohol	4-Ethylphenol	1904	1987	50-5000	0.9991
Alcohol	4-Vinylguaicol	1725	1720	50-5000	0.9960
Alcohol	4-Vinylphenol	2439	2415	50-5000	0.9917
Alcohol	3-Ethoxy-1-propanol	1377	1380	50-5000	0.9960
Alcohol	*trans*-3-Hexen-1-ol	1682	1690	50-5000	0.9970
Alcohol	*cis*-3-Hexen-1-ol	1713	1725	50-5000	1.0000
Alcohol	Isoamyl alcohol	1206	1211	50-5000	0.9994
Alcohol	3-Methyl-3-pentanol	1080	1074	50-5000	0.9901
Alcohol	4-Ethylguaiacol	2030	2035	50-5000	0.9953
Alcohol	Isohexanol	1311	1316	50-5000	0.9999
Alcohol	Benzyl Alcohol	1860	1851	50-5000	0.9992
Alcohol	Isobutanol	1093	1097	50-5000	0.9987
Alcohol	Phenylethyl Alcohol	2199	2203	50-5000	1.0000
Alcohol	2-methyl-1-butanol	1199	1123	50-5000	0.9991
Alcohol	4-methyl-1-pentanol	1336	1321	50-5000	0.9944

Alcohol	2-penten-1-ol	1335	1347	50-5000	0.9989
Alcohol	Furfuryl alcohol	1688	1695	50-5000	0.9917
Alcohol	Benzylalcohol	1906	1931	50-5000	0.9944
C13	*β*-Damascenone	1821	1834	5-300	0.9960
C13	TDN	1714	1719	5-300	0.9997
C13	α- Ionone	1877	1869	5-300	0.9996
C13	β-Ionone	1912	1901	5-300	0.9998
Ester	Isobutyl acetate	1032	1019	50-5000	0.9959
Ester	2-Phenylethyl acetate	1813	1820	50-5000	0.9978
Ester	*trans*-3-Hexen-1-yl acetate	1309	1300	50-5000	0.9971
Ester	*cis*-3-Hexen-1-yl acetate	1327	1333	50-5000	0.9975
Ester	Isoamyl acetate	1126	1136	50-5000	0.9960
Ester	Isoamyl decanoate	1851	1865	50-5000	0.9957
Ester	Diethyl malate	1235	1240	50-5000	1.0000
Ester	Diethyl succinate	1699	1709	50-5000	0.9999
Ester	Ethyl 2-hydroxy-4-methylpentanoate	1515	1529	50-5000	1.0000
Ester	Ethyl 2-hydroxy-3-methylbutanoate	1067	1059	50-5000	0.9971
Ester	Ethyl butanoate	1053	1028	50-5000	0.9912
Ester	Ethyl lactate	1347	1356	50-5000	0.9897
Ester	Ethyl 3-hydroxyhexanoate	1661	1656	50-5000	0.9953
Ester	Ethyl 3-hydroxytridecanoate	2433	2426	50-5000	0.9950
Ester	Ethyl 9-decenoate	1703	1710	50-5000	0.9925
Ester	Ethyl 9-hexadecenoate	2269	2261	50-5000	0.9894
Ester	Ethyl decanoate	1606	1625	50-5000	0.9931
Ester	Ethyl dodecanoate	1811	1824	50-5000	0.9913
Ester	Ethyl heptanoate	1352	1341	50-5000	0.9908
Ester	Ethyl hexanoate	1239	1246	50-5000	0.9933
Ester	Ethyl hydrogen succinate	2367	2375	50-5000	0.9886
Ester	Ethyl nonanoate	1530	1555	50-5000	0.9956
Ester	Ethyl octanoate	1435	1430	50-5000	0.9933
Ester	Ethyl oleate	2461	2469	50-5000	0.9912
Ester	Ethyl furoate	1648	1667	50-5000	0.9933
Ester	Ethyl palmitate	2246	2240	50-5000	0.9929
Ester	Hexyl acetate	1279	1291	50-5000	0.9960
Ester	Methyl octanoate	1399	1409	50-5000	0.9907
Ester	Methyl salicylate	1755	1746	50-5000	0.9954
Ester	Ethyl vanillate	1425	1446	50-5000	0.9911
Ester	Ethyl linoleate	2645	2658	50-5000	0.9950
Terpene	1,8 Terpin	2122	2105	5-300	0.9941
Terpene	Citronellyl acetate	1657	1666	5-300	0.9881
Terpene	Farnesol	2380	2369	5-300	0.9939

Terpene	4-Carene	1134	1130	5-300	0.9958
Terpene	allo-Ocimene	1367	1370	5-300	0.9957
Terpene	Ocimenol	1669	1687	5-300	0.9918
Terpene	*α*-Ocimene	1232	1235	5-300	0.9938
Terpene	*α*-Pinene	1110	1104	5-300	0.9932
Terpene	*α*-Terpinene	1179	1185	5-300	0.9926
Terpene	*α*-Terpineol	1694	1710	5-300	0.9969
Terpene	*α*-Terpinolene	1280	1568	5-300	0.9946
Terpene	*β*-Ocimene	1235	1240	5-300	0.9960
Terpene	*β*-Pinene	1110	1104	5-300	0.9959
Terpene	*cis*-*α*-Bisabolene	1775	1783	5-300	0.9935
Terpene	*cis*-*β*-Farnesene	1664	1671	5-300	0.9904
Terpene	*trans*-*β*-Farnesene	1660	1648	5-300	0.9954
Terpene	*cis*-Linalool oxide (furanoid)	1440	1500	5-300	0.9918
Terpene	Citronellol	1764	1771	5-300	0.9898
Terpene	Geranyl formate	1686	1694	5-300	0.9911
Terpene	Geranyl acetate	2334	2374	5-300	0.9980
Terpene	Hotrienol	1603	1567	5-300	0.9943
Terpene	*β*-Caryophyllene	1599	1609	5-300	0.9959
Terpene	Limonene	1198	1209	5-300	0.9922
Terpene	Linalool	1543	1557	5-300	0.9939
Terpene	Linalyl isobutyrate	1622	1602	5-300	0.9949
Terpene	Menthol	1630	1624	5-300	0.9970
Terpene	p-Cymene	1270	1265	5-300	0.9963
Terpene	Tetrahydrolinallol	1420	1439	5-300	0.9955
Terpene	8-hydroksylinalool	2339	2351	5-300	0.9899
Terpene	Linalyl format	1487	1466	5-300	0.9928
Terpene	Terpinen-4-ol	1624	1611	5-300	0.9947
Terpene	β-ionone-5,6-epoxide	2011	2001	5-300	0.9905
Terpene	Menthol	1660	1679	5-300	0.9946
Terpene	Neric acid	2381	2354	5-300	0.9977
Terpene	Nerol	1822	1806	5-300	0.9982
Terpene	Nerolidol	2059	2034	5-300	0.9917
Terpene	2,6-Dimethyl-3,7-octadien-2,6-diol	1718	1829	5-300	0.9925
Terpene	6,7-dihydro-7-hydroxylinalool	1985	1972	5-300	0.9916
Terpene	2,6-dimethyl-7-octen-2,6-diol	1995	2009	5-300	0.9958
Terpene	Geraniol	1868	1884	5-300	0.9953
Terpene	Terpendiol II	1965	1949	5-300	0.9971
Terpene	Ethyl linalyl acetate	1480	1428	5-300	0.9966
Lactone	*cis*-Whiskey lactone	1993	1981	5-300	0.9952
Lactone	*δ*-Nonalactone	2235	2249	5-300	0.9958

Lactone	*γ*-Butyrolactone	1666	1699	5-300	0.9960
Lactone	*γ*-Decalactone	2183	2201	5-300	0.9944
Lactone	*γ*-Hexalactone	1738	1887	5-300	0.9959
Lactone	*γ*-Nonalactone	2067	2100	5-300	0.9957
Lactone	*γ*-Octalactone	1953	1972	5-300	0.9951
Lactone	γ-Undecalactone	2299	2305	5-300	0.9957
Lactone	trans-Whiskey lactone	1921	1947	5-300	0.9954
Aldehydes	2,4-hexadienal	1398	1405	50-5000	0.9948
Aldehydes	Benzaldehyde	1553	1568	50-5000	0.9979
Aldehydes	*trans*-2,4-heptadienal	1518	1531	50-5000	0.9965
Aldehydes	Decanal	1518	1511	50-5000	0.9963
Aldehydes	Acetylfuran	1533	1517	50-5000	0.9924
Aldehydes	2,4-nonadienal	1728	1701	50-5000	0.9959
Aldehydes	2,4-decadienal	1837	1815	50-5000	0.9961
Aldehydes	*cis*-2,4-heptadienal	1488	1497	50-5000	0.9982
Aldehydes	Furfural	1492	1506	50-5000	0.9970
Phenols	Homovanillyl alcohol	2817	2805	50-5000	0.9928
Phenols	Eugenol	2205	2221	50-5000	0.9963
Phenols	Vanillin	2577	2539	50-5000	0.9980
Other compounds	2-pentylfuran	1247	1223	50-5000	0.9936
Other compounds	Acetoin	1307	1332	50-5000	0.9925
Other compounds	6-methyl-5-hepten-2-one	1340	1329	50-5000	0.9977
Other compounds	4-ethyl-cyclohexanol	1518	1529	50-5000	0.9967
