# Supplementary material for: Sequential Fermentation in Red Wine cv. Babić Production: The Influence of Torulaspora delbrueckii and Lachancea thermotolerans Yeasts on the Aromatic and Sensory Profile
Source: Foods. 2024 Jun 25;13(13):2000. doi: 10.3390/foods13132000 (PMC11241832; doi:10.3390/foods13132000)
Supplement: Supplementary file 1 [file foods-13-02000-s001.zip › foods-3051206-Table S2.pdf]

Supplemental Table S2. Sensory attributes and material used for the sensory panel training

Attribute	Definition	Material
Fruity	Overall intensity of fruit aromas; raspberry, blackcurrant, blackberry, cherry, plum	Aromaster
Dry fruits	Intensity of the aroma of dried fruits; raisins, prune	Aromaster
Floral	Intensity of the aromas of fresh or dried flowers such as roses, acacia, lavender, violet	Aromaster
Vegetal	Intensity of the aroma of cut grass, fresh grass, leaves, capsicum	Aromaster
Nutty	Intensity of the flavors of nuts; hazelnut, almond	Aromaster
Herbs	Intensity of mixed aromatic herbs such as thyme, clove, liquorice	Aromaster
Other/ Cheesy, buttery	Intensity of the aroma of cheese and/or sweat	0.1 g/L Diacetyl (Sigma) in base wine
Other / Earthy	Intensity of the aromas of mushroom, tree moss, truffle	Aromaster
Other / Woody	Intensity of the aroma oak, cedar, smoke	0.3 g/L French oak chips (AEB) in base wine
Bitterness	Intensity of bitter taste perceived in the mouth, including after expectorating, noticed at the back of the throat	12 mg/L quinine sulphate in aqueous solution
Acidity	Intensity of acid taste perceived in the mouth, including after expectorating, sharp taste	1.5 g/L tartaric acid in aqueous solution
Aftertaste	The taste intensity of a wine perceived immediately after the wine is removed from the mouth.	
Fullness / body	Sensation in the mouth in which all of the attributes or tactile sensations are added, describes the lightness or fullness of the wine in the mouth	
Flavor quality	Spectrum of properties and characteristics of a wine that gives an aptitude to satisfy, nose and taste, implicit or expressed needs	
Harmony	Interrelationship between all the taste sensations and the components that create them in the mouth	
Overall impression	Evaluation of all olfactory and gustatory attributes presented in wine sample	
